# Blood Will Tell: What Hematological Analyses Can Reveal About Fish Welfare

**DOI:** 10.3389/fvets.2021.616955

**Published:** 2021-03-30

**Authors:** Henrike Seibel, Björn Baßmann, Alexander Rebl

**Affiliations:** ^1^Institute of Animal Breeding and Husbandry, Christian-Albrechts-University, Kiel, Germany; ^2^Gesellschaft für Marine Aquakultur mbH (GMA), Büsum, Germany; ^3^Department of Aquaculture and Sea-Ranching, Faculty of Agricultural and Environmental Science, University of Rostock, Rostock, Germany; ^4^Institute of Genome Biology, Leibniz Institute for Farm Animal Biology (FBN), Dummerstorf, Germany

**Keywords:** erythrocytes, hematology, leukocytes, teleost fishes, well-being, transcriptomics, proteomics, stress

## Abstract

Blood analyses provide substantial information about the physiological aspects of animal welfare assessment, including the activation status of the neuroendocrine and immune system, acute and long-term impacts due to adverse husbandry conditions, potential diseases, and genetic predispositions. However, fish blood is still not routinely analyzed in research or aquaculture for the assessment of health and/or welfare. Over the years, the investigative techniques have evolved from antibody-based or PCR-based single-parameter analyses to now include transcriptomic, metabolomic, and proteomic approaches and from hematological observations to fluorescence-activated blood cell sorting in high-throughput modes. The range of testing techniques established for blood is now broader than for any other biogenic test material. Evaluation of the particular characteristics of fish blood, such as its cell composition, the nucleation of distinct blood cells, or the multiple isoforms of certain immune factors, requires adapted protocols and careful attention to the experimental designs and interpretation of the data. Analyses of fish blood can provide an integrated picture of the endocrine, immunological, reproductive, and genetic functions under defined environmental conditions and treatments. Therefore, the scarcity of high-throughput approaches using fish blood as a test material for fish physiology studies is surprising. This review summarizes the wide range of techniques that allow monitoring of informative fish blood parameters that are modulated by different stressors, conditions, and/or treatments. We provide a compact overview of several simple plasma tests and of multiparametric analyses of fish blood, and we discuss their potential use in the assessment of fish welfare and pathologies.

## Introduction

A significant segment of the constantly growing aquaculture sector is represented by intensive farming practices (1) aimed at meeting the increasing demand of a growing world population. However, at the same time, this emphasis raises many concerns due to the competition for resources and the negative impact of intensive aquaculture on the environment and fish welfare (2). Consequently, the visionary concepts for future fish farming include welfare-friendly systems that can monitor animal-based parameters and quickly regulate disruptive environmental variables when necessary (3, 4). Certified farming procedures should be based on a comprehensive, science-based understanding of how teleost fish respond to anthropogenic environmental disruptions and challenging aquaculture-related conditions. This requires multidisciplinary investigations to define and evaluate optimal species-specific husbandry conditions, especially for newly introduced aquaculture species (5). Consequently, research groups and large scientific consortia are currently studying the influences of a variety of factors, ranging from global climate change, ocean acidification, and eutrophic habitats to introduced pathogens and pollution by environmental toxins or microplastics, on fish breeding and wild fish stocks. The use of more extensive research approaches to screen the effects of environmental conditions can increase the probability of detecting disturbances and even dangerous confounding factors, thereby allowing the discovery of relevant diagnostic biomarkers for fish health.

One favorable option for rapid and non-lethal sampling of large numbers of fish individuals is blood analysis. Blood is a complex mixture of heterogeneous cell populations (6–8) that include erythrocytes (red blood cells), leukocytes (white blood cells), and thrombocytes (analogs of mammalian platelets) (9). These fish blood cells are broadly similar in function to their mammalian counterparts and are found in all other tissues and organs throughout the body. Blood transports an immense variety of substances (gases, water, minerals, nutrients, hormones, immune effectors, toxins, microbial structures, or waste products), so its analysis can provide a wealth of information about fish physiology and health status. Alterations in informative blood-based indicators like metabolite concentrations, hormonal profiles, and transcript abundances can reflect systemic reactions to changes or disturbances in homeostasis that can alert scientists and veterinarians (as well as fish farmers), who monitor the physiological status of an individual fish or an entire population.

**Figure 1:**
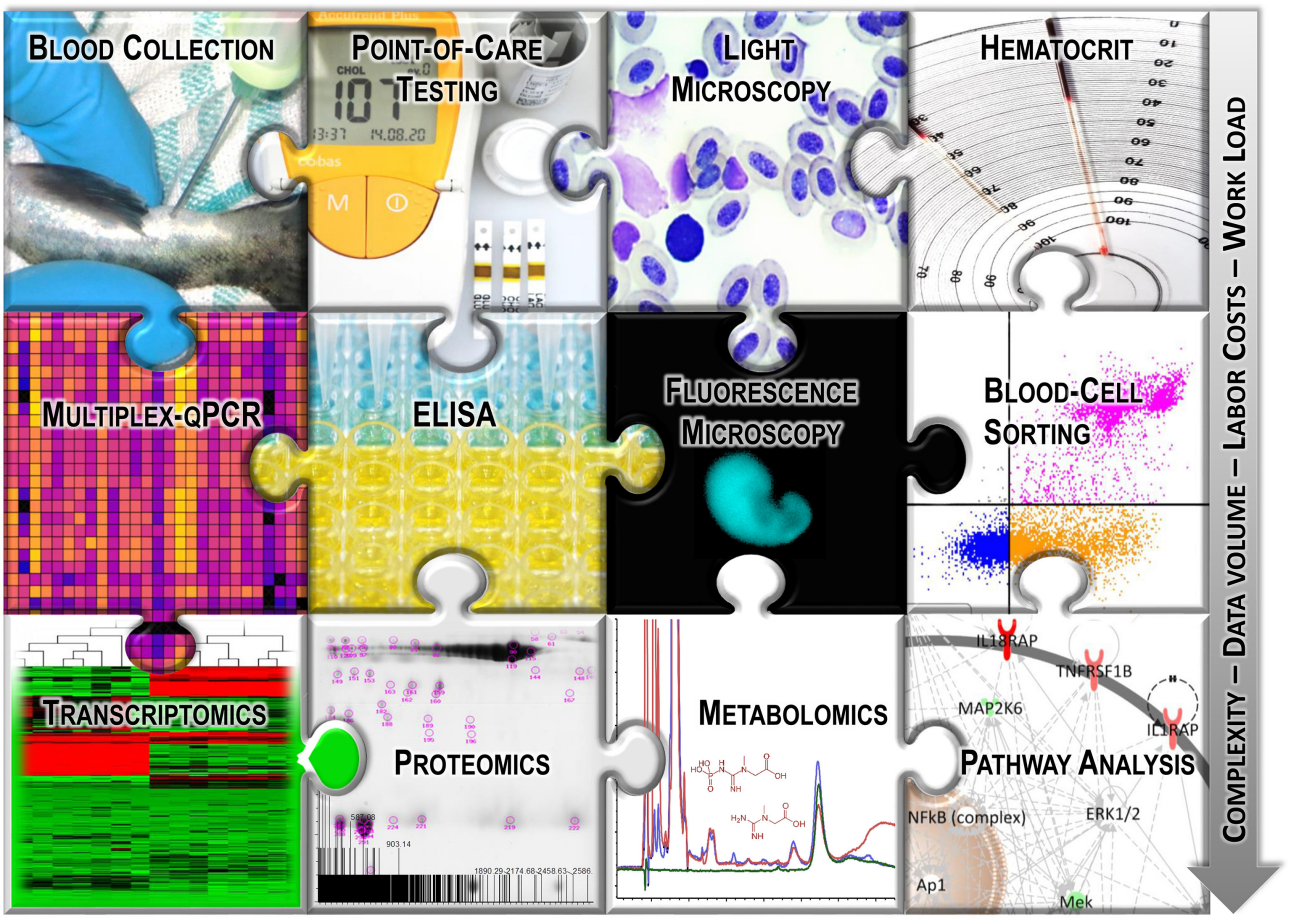
Graphical overview of the selected analysis techniques used with fish blood.

Blood tests on fish have been carried out for decades (10) in laboratory and field settings to assess endocrine, reproductive, and immune functions; maturation; nutrition and health status or to perform genetic studies (11). More analysis techniques have been established for blood as a test material (Figure 1) than for any other tissue or fluid sample. Nevertheless, interpreting the obtained data to extract meaningful information on the individual condition remains difficult. On the one hand, a few excellent reviews have recently updated the history of selected hematological techniques (12, 13); however, remarkably, blood analyses using systems biological approaches or even PCR-based techniques are still underrepresented in fish physiology (14). On the other hand, the omics-based hypotheses put forward by fish geneticists and molecular biologists are correlated only on a small scale with non-transcript parameters extracted from fish blood.

A growing part of the research on farmed fish focuses on “animal welfare” involving optimization of husbandry conditions, stress avoidance, and improvement of fish quality of life (15, 16). The focus is largely on the ability of domesticated fish populations to cope with environmental and/or anthropogenic challenges (17). In general, animal-welfare programs aim to ensure freedom from (i) thirst, hunger, and malnutrition; (ii) discomfort; (iii) pain, injury, and disease; (iv) restriction of normal behaviors; and (v) fear and suffering (18). These basic aspects also apply to some extent to the welfare of fish in aquaculture. However, fish require different treatment than terrestrial animals in many ways due to their aquatic nature and differences in their anatomy and physiology, as well as in their required husbandry conditions. According to Huntingford et al. (19), fish welfare can be expressed by (i) a feelings-based definition, focusing on a reduction in pain and negative emotions, and/or increased access to positive experiences; (ii) a nature-based definition assuming that every fish species must express its inherent biological nature; or (iii) a function-based definition targeting the ability of fish to adapt to environmental demands.

The well-being of fish in aquaculture is impaired by acute environmental changes, coupled with husbandry practices, such as sorting, grading, and transport that can induce stress responses. Most research studies are based on the third concept (iii), which involves fish health and the adequate functioning of fish biological systems, especially those involved in managing a compromised homeostasis imposed by aquaculture conditions. The physiological state of a fish with disturbed homeostasis is usually captured by observing and recording indicative signs and measurable characteristics of the response to environmental challenges or stressors. Restoration of homeostasis usually requires the invocation of complex behavioral and physiological adaptive responses (20).

Primary stress responses are characterized by the release of catecholamines and corticosteroids (21). The subsequent secondary stress responses have a multitude of actions in many tissues, including blood, and can range from accelerated mobilization of energy *via* glucose, altered hydromineral balance, and increased lactate levels to decreased blood pH, hematocrit, and sodium levels and lower liver glycogen levels (20, 21). Increased cardiovascular activity and breath rate that enhance the uptake and transport capacity for oxygen are accompanied by a redistribution or suppression of immune functions (21, 22). Tertiary responses are reflected by behavioral adaptions, inhibited growth, decreased reproduction, and a compromised capacity to endure any additional stressors. The repeated exposure to a low-intensity stressor triggers the development of an adaptive response over time and attenuates acute stress reactions, whereas the exposure to harmful, persistent stressors is likely to intensify the physiological response (23). When stressors co-occur with a high pathogenic pressure, they provoke serious diseases (24) and strongly impair fish welfare (25).

Many previous and current studies on fish welfare have measured the main components of the primary physiological stress response, largely plasma cortisol and glucose (26–28). Both components are informative stress markers, but they have limitations (29). The current scientific consensus is that the assessment of fish welfare is a complex task (30), in part because the absence of a physiological stress response does not necessarily imply adequate welfare (31). Each fish species has distinct “ecological and behavioral demands” (15); therefore, the responses to adverse conditions vary across taxa (32, 33). In recent years, the scientific evaluation of fish physiology has shifted from the conventional, limited biomarker approach to comprehensive and rather holistic approaches (34, 35). The spectrum of the parameters now recorded has expanded and is accompanied by an increasingly well-equipped fish-specific toolbox (Figure 1). These advances now facilitate the generation of weighted welfare scores (36, 37) that can integrate operational and laboratory-based parameters.

Laboratory data, such as gene expression studies (30, 38, 39), are based on RNA specimens that are mostly obtained from the organs of previously killed fish. By contrast, fish blood collection can be conducted non-lethally (11); therefore, blood represents an alternative and preferable matrix for ethical reasons. Though relatively non-invasive, blood sampling is generally stressful for fish, but repeated blood sampling from the same individual provides the possibility of tracking the time course of various processes after the treatment or determining fish welfare during the developmental stages. Other operational parameters, such as the monitoring of exploratory and swimming behavior (40, 41) or the rate of gill ventilation (42), can readily be recorded on commercial farms, even those that lack the well-equipped wet laboratories.

This review compiles the most commonly reported techniques for obtaining chemico-physical, cellular, metabolic, and transcriptional information from fish blood. It also provides interpretations of the various methods in terms of their importance in assessing the impact of changing environmental conditions or experimental treatments and the intensity and duration of the response of fish.

## Anesthesia and Blood Sampling Procedures

For gentle handling and for safety reasons, fish should be sedated prior to and during blood sampling to minimize pain or discomfort and to prevent defensive or flight reactions and subsequent injuries. An analgesic alone may mask the sensation of pain but will not prevent the perception of subsequent treatments. A loss of consciousness, therefore, ensures the welfare of the fish during blood collection. The most commonly used anesthetic is MS-222 (also known as Tricaine-S or 3-ethoxycarbonylanilinium methanesulfonate) (43), although clove oil (containing eugenol, 4-allyl-2-methoxyphenol), quinaldine (2-methylquinoline), 2-phenoxyethanol, and benzocaine (ethyl 4-aminobenzoate) are also commonly used to stun fish (44). The legal provisions of a given country determine the circumstances that justify the use of particular fish anesthetics. Anesthetics are usually administered as an immersion bath, but the capture of the fish and its transfer to the anesthetic bath, with the brief exposure to air, are likely to evoke a stress reaction (45, 46). Therefore, these steps should be carried out quickly. The anesthetic *per se* can also act as a stressor on the individual fish being treated.

MS-222 blocks the sodium channels and action potentials of neurons, but concentrations of up to 300 mg MS-222 per liter were shown to have no significant impact on the plasma cortisol levels of South American silver catfish (*Rhamdia quelen*) (47). By contrast, gilthead sea bream (*Sparus aurata*) showed significantly increased cortisol levels following the exposure to even 25 mg/L MS-222 or 0.075 mg/L 2-phenoxyethanol (48). In zebrafish, 15 mg/L of buffered MS-222 altered various hematological characters, including hematocrit, coagulation, and the amount of blood collected (49). The influence of a given anesthetic on the biochemical profile of a sample obviously depends on the dose and on the treated species. These considerations should therefore be taken into account during the evaluation of fish blood parameters. Nevertheless, the anesthesia certainly facilitates the blood collection and prevents a more pronounced stress response, so these advantages may outweigh the potential difficulties related to stress diagnostics.

**Figure 2:**
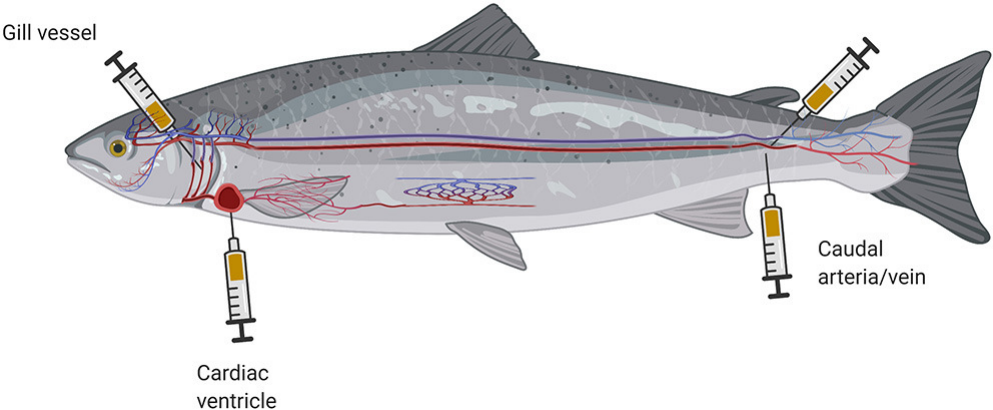
Schematic illustration of different cannula placements for blood collection from fish (created with BioRender.com).

Optimal blood collection depends on the size and anatomical characteristics of the investigated individual (11). The least traumatic and most widely used blood sampling procedure is to withdraw blood from the caudal vessels—laterally or ventrally—using a cannula and syringe filled with an anticoagulant (sodium citrate, heparin, or potassium ethylenediaminetetraacetic acid) (Figure 2). Some analyses require quite large amounts of blood, which creates difficulties when the blood is collected from small teleosts with an estimated whole-blood volume of 3–7 ml per 100 g (50–52). Fish smaller than 8 cm in length are difficult to bleed, but one option is to sever the fin of an anesthetized small fish and then centrifuge the fish at low force (14). Larger teleosts may also be bled by puncture of the cardiac ventricle or other vessels, such as gill-blood vessels (Figure 2) (53).

These blood sampling techniques are reserved for studies aimed at answering distinct research questions (54), as they bear a high risk of late complications. In this context, a point worth noting is that the site of blood collection often influences the obtained biomarker profile (55). More detailed information on blood collection techniques has been provided in recent review articles (45, 56). After blood sampling, the fish should be transferred to an aerated recovery tank to allow it to regain perception, awareness, and ability to react. Immediate return of a fish to its usual environment poses a risk that it may be attacked by its tank mates if it remains in a state of non-reactivity.

The liquid part of the blood—the plasma fraction—transports diverse metabolites of interest, including hormones and catabolic products, such as proteins, amino acids, glucose, lipids, and organic acids. For this reason, many researchers separate the plasma *via* centrifugation with an anticoagulant or obtain the serum after blood clotting and subsequent centrifugation (57). Hence, plasma contains natural coagulation factors, whereas serum contains all plasma components except the coagulation factors. Thus, the choice of using plasma or serum depends largely on the research question and its related need for coagulation factors.

## Immunoassays and Clinical Test Kits

Many studies that have evaluated fish welfare in aquaculture have revealed that aversive husbandry conditions alter the stress-hormone levels in the farm fish. Stressors and adverse conditions can trigger the chromaffin cells of the head kidney to rapidly release catecholamines, such as noradrenaline/norepinephrine and adrenaline/epinephrine. These reactions may also occur in direct response to the catching process, the subsequent anesthesia, and/or the blood sampling, so they complicate the evaluation of experimentally induced challenges. However, the hypothalamus generates a parallel production of corticotropin-releasing hormone (CRH), which then stimulates the pituitary gland to release adrenocorticotropic hormone (ACTH) (19, 58, 59). This is followed by the synthesis and secretion of cortisol by the interrenal tissue in the head kidney. Many scientists prefer to measure cortisol or ACTH as blood-based indicators of acute stress responses due to the slow increase (within minutes) of these compounds.

Cortisol is probably the most commonly measured molecule for determining the effects of stress in fish (16, 35). Cortisol and ACTH are commonly measured using radioimmunoassays (RIAs). Typically, RIA incorporates a radioactive iodine isotope due to its decay properties. However, the higher technical effort required in an isotope laboratory has led to an increasing preference for ELISAs (60) as an alternative non-radioactive method for the cortisol and ACTH measurements. Both RIAs and ELISAs are antibody-based detection methods, but ELISAs use specific antibodies that bind to the substance (antigen) and rely on an enzymatic color reaction for antigen detection. Electrochemical immunoassays (61), gas or liquid chromatography (62), mass spectrometry (63), and fluorescence-based methods (64) are alternatives to RIA and ELISA for determining blood cortisol levels.

Increased cortisol levels stimulate a number of metabolic processes, such as glucogenolysis and gluconeogenesis, to mobilize and allocate energy reserves (e.g., glucose release into the bloodstream) (21). Increased, as well as decreased, glucose levels are considered signs of stress (Table 1) (88), as stress-induced alterations in muscle activity accelerate the conversion of glucose to lactate (or alternatively to pyruvate) by anaerobic glycolysis. The resulting plasma levels of lactate and glucose are often used in conjunction with cortisol levels to establish the physiological state and to assess the severity of a stress response (9, 87, 99–101). Some studies have demonstrated that even the removal of an individual *S. aurata* fish evoked an acute stress reaction in the remaining fish in the tank. The plasma cortisol levels in the tank fish returned to the initial level after 8 h, whereas the plasma glucose and lactate levels and other hematological parameters required 24 h for recovery (102). The plasma triglycerides or cholesterol levels can also reflect the metabolic changes and serve as physiological indices (86), as these lipids can be used as alternate sources of energy by the fish (103–105). Blood glucose, lactate, and triglyceride levels can be determined easily and quickly using appropriate cuvette tests or test strips in portable devices designed for clinical test kits (also known as point-of-care tests) (76). The advantages of point-of-care tests are their time-saving and simple operation, with their reliable and easily readable results.

**Table 1:** Overview of the selected physiological parameters that change in blood, plasma, or serum in response to stressful conditions in various fish species.

**Stressor/condition**	**Species^**a**^**	**Duration of challenge**	**Putative indicator**	**Direction**	**References**
**Handling:**					
Transportation	*I. punctatus*	20 min	Circulating granulocytes and T cells	Up	(65)
Transportation	*I. punctatus*	20 min	Circulating B cells	Down	(65)
Catching, sorting, transportation	*R. rutilus, A. brama*	3 h	Sodium-ion contents	Down	(66)
Handling (15 s)	*S. salar*	2 weeks	O-phosphocholine, lactate, carbohydrates, alanine, valine, trimethylamine-N-oxide	Up	(67)
Handling (15 s)	*S. salar*	4 weeks	Di-O-acetylated sialic acids	Up	(68)
Transfer to another tank	*S. trutta*	1 min	Cortisol, glucose, lactate	Up	(69)
Aerial exposure	*H. sabinus*	30 min	Lactate, acidosis, glucose	Up	(9)
Aerial exposure	*S. senegalensis*	3 min	Cortisol, glucose, lactate, osmolality levels	Up	(70)
“Hook and line” stress^b^	*O. mykiss*	2 min	Cell counts, hematocrit, glucose, thrombocytes	Up	(71)
“Hook and line” stress^b^	*O. mykiss*	2 min	Clotting time	Down	(71)
Manual stripping procedure	*O. mykiss*	5 days	Cortisol, *TNF*	Up	(72)
**Temperature**					
16°C	*G. morhua*	5 days	*B2M, MHC-I, IGL* mRNA	Up	(73)
20°C	*C. catla*	12 h	*TLR2,−4,−5* and *NOD1,−2* mRNA	Up	(74)
23°C	*D. labrax*	12 h	Cortisol, glucose, superoxide dismutase activity	Up	(75)
30°C	*P. mesopotamicus*	5 days	Glucose	Up	(76)
35°C	*C. catla*	12 h	*TLR2,−4*, and *NOD1,−2* mRNA	Up	(74)
36°C	*R. holubi*	7 days	Cortisol	Up	(28)
37°C	*E. coioides*	1 h	Immunoglobulin M	Up	(77)
**Oxygen saturation**					
0.3 ppm	*O. niloticus*	3 days	Heat-shock protein 70	Up	(78)
1.0 ppm	*C. catla*	1 h	*HMBG1, TLR4, MYD88, NOD1, RICK, IL6, CXCL8, IL10* mRNA	Up	(79)
1.3 ppm	*S. aurata*	>11 h	Hematocrit, hemoglobin, glucose, lactate	Up	(80)
1.3 ppm	*S. aurata*	>11 h	*UCP2* mRNA	Down	(80)
1.3 ppm	*S. aurata*	4 h	*GST3, UCP2, ATPAF2, SCO1, MIRO1a, TIM44, TIM10, ACAA2* mRNA	Down	(81)
1.3 ppm	*S. aurata*	4 h	Glucose, lactate, hematocrit, hemoglobin	Up	(81)
2.3 ppm coupled with stocking of 19 kg/m^3^	*S. aurata*	22 days	*NDUFAF2* mRNA	Down	(82)
2.3 ppm coupled with stocking of 9.5 kg/m^3^	*S. aurata*	22 days	Hematocrit, growth hormone, lactate, erythrocyte number	Up	(82)
**Stocking:**					
30 kg/m^3^	*S. salar*	10 weeks	Alkaline phosphatase	Up	(83)
30 kg/m^3^	*S. salar*	10 weeks	Immunoglobulin M	Down	(83)
30 kg/m^3^	*S. salar*	10 weeks	Cortisol	Up	(83)
30 kg/m^3^	*S. salar*	10 weeks	Maleic dialdehyde	Up	(83)
34 kg/m^3^	*S. aurata*	15 weeks	Cortisol, plasma proteins, hematocrit, hemoglobin, erythrocyte number	Up	(84)
40 kg/m^3^, 80 kg/m^3^	*O. mykiss*	9 months	Cortisol	Down	(85)
45 kg/m^3^	*O. mykiss*	1 month	Cholesterol	Down	(86)
45 kg/m^3^	*O. mykiss*	1 month	Glucose	Up	(86)
45 kg/m^3^	*O. mykiss*	1 month	Triglyceride	Down	(86)
70 kg/m^3^	*O. mykiss*	2 days	Lactate	Up	(87)
100 kg/m^3^	*C. maraena*	9 days	Circulating myeloid cells	Up	(32)
100 kg/m^3^	*C. maraena*	9 days	Circulating thrombocytes	Down	(32)
120 kg/m^3^	*S. fontinalis*	1 month	Glucose	Down	(88)
**Nutrition:**					
Food deprivation	*O. mykiss*	28 days	Very-low-density lipoproteins	Up	(89)
Food deprivation	*O. mykiss*	28 days	High-density lipoprotein, choline, β-glucose, lactate	Down	(89)
Plant-based diet with yeast fraction	*O. mykiss*	84 days	Histidine	Down	(90)
Food supplementation with menthol oil	*O. niloticus*	15 days	Hematocrit, erythrocytes, leukocytes, globulin and albumin content, protein concentration, lysozyme and phagocytic activity	Up	(91)
Food supplementation with roselle powder	*O. mykiss*	60 days	Erythrocytes, hematocrit, activities of superoxide dismutase and catalase	Up	(92)
**Pollution**					
Metallic/organic compounds	*D. labrax*	15 days	Glucose, cortisol, superoxide dismutase activity	Up	(75)
Oxytetracycline	*O. mykiss*	14 days	Sodium dismutase, erythrocyte and leukocyte number	Down	(93)
Nitrite	*P. fulvidraco*	4 days	Sodium- and chloride-ion contents, erythrocyte number, hemoglobin, total antioxidant capacity, activities of superoxide dismutase and catalase and glutathione peroxidase	Down	(94)
Nitrite	*P. fulvidraco*	4 days	Sodium- and chloride-ion contents, leukocyte number, malondialdehyde content	Up	(94)
Polystyrene nanoplastics	*C. idella*	20 days	Erythrocyte nuclear abnormalities, altered erythrocyte morphometry	Up	(95)
**Other environmental conditions**					
Low-dose ultraviolet B radiation	*C. carpio*	6 weeks	Total protein concentration, oxidative burst activity	Down	(96)
Low-dose ultraviolet B radiation	*O. mykiss*	6 weeks	Oxidative burst activity, cortisol, lymphocyte number	Up	(96)
High CO_2_ levels	*H. hippoglossus*	14 weeks	Complement C3, fibrinogen	Up	(97)
Low salinity	*D. labrax*	12 h	Glucose, cortisol, hemoglobin, peroxidase and superoxide-dismutase activity	Up	(75)
Open field (absence of shelter)	*B. episcopi*	2 min	Cortisol	Up	(98)

a *Abramis brama (A. brama), Brachyrhaphis episcopi (B. episcopi), Catla catla (C. catla), Coregonus maraena (C. maraena), Ctenopharyngodon idella (C. ide), Cyprinus carpio (C. carpio), Dicentrarchus labrax (D. labrax), Epinephelus coioides (E. coioides), Gadus morhua (G. morhua), Hippoglossus hippoglossus (H. hippoglossus), Hypanus sabinus (H. sabinus), Ictalurus punctatus (I. punctatus) Oncorhynchus mykiss (O. mykiss), Oreochromis niloticus (O. niloticus), Pelteobagrus fulvidraco (P. fulvidraco), Piaractus mesopotamicus (P. mesopotamicus), Rhabdosargus holubi (R. holubi), Rutilus rutilus (R. rutilus), Salvelinus fontinalis (S. fontinalis), Salmo salar (S. salar), Salmo trutta (S. trutta), Solea senegalensis (S. senegalensis), Sparus aurata (S. aurata)*.

b *Hook insertion into the caudal peduncle forcing fish to swim for 2 min by applying tension to the line*.

Changes in cortisol levels indicate the magnitude of a primary stress response, but usually do not provide sufficient information to gauge the ability of an animal to cope with a challenging condition (106). Notably, cortisol levels can vary widely among individuals in response to diurnal or seasonal fluctuations and the ambient temperature (107–109) or according to gender and maturity (58, 106, 110, 111). The cortisol level depends on many factors that can have diverse interactions (29). In optimal cases, the cortisol level rises in correlation with the intensity of a stressor and returns to its baseline level if the stressor does not persist. Multiple stressors occurring simultaneously or persistent chronic stress conditions can complicate the interpretation of a measured cortisol level, as cortisol release might be suppressed by feedback interactions through the activated stress axis. In particular, differences in treatments can complicate comparisons between experiments (112); therefore, we recommend measuring other parameters of the secondary and tertiary stress response to establish the ability of the animal to cope with stressors and to assess its well-being.

## Hematological Profiling and Blood Cell Sorting

The original technique for obtaining a differential blood count is simple and relatively inexpensive, but time-consuming and tedious (113). Hematological evaluations are usually based on blood smears and require a sound knowledge of blood cell morphology.

The vast majority of blood cells are erythrocytes, which ensure a sufficient supply of oxygen in the various body tissues. Metabolic alterations associated with physical work, excitement, and stress responses increase the tissue oxygen requirements, so large numbers of erythrocytes are additionally recruited and mobilized from depots in the spleen (114). For instance, rainbow trout (*Oncorhynchus mykiss*) responded to a 2-month food supplementation with roselle (*Hibiscus sabdariffa*) meal by significantly increasing the number of erythrocytes in the blood and simultaneously lowering the blood cortisol and glucose levels (92). By contrast, Indian major carp (*Labeo rohita*) exposed to water 6°C warmer than usual showed significantly increased glucose levels and a reduction in erythrocyte counts (115). A recent examination of the erythrocytes from grass carp (*Ctenopharyngodon idella*) did not reveal any significant alteration in numbers after 20 days of exposure to polystyrene nanoplastics, but numerous abnormalities were observed regarding the shape and size of the cells and their nuclei (95).

Adverse environmental conditions can affect the numbers and shapes of circulating erythrocytes, but they can also change the composition of leukocytes in circulation (58, 116, 117). Leukocytes are generally subdivided into monocytes, lymphocytes, and granulocytes (118). Granulocyte staining with Romanowsky/Wright or May–Grünwald–Giemsa stains can differentiate the eosinophilic, basophilic, and neutrophilic types (119, 120) that occur in tetrapods. Some fish species possess heterophilic granulocytes that are characterized by the presence of additional eosinophilic granules (121, 122).

As reported in other vertebrates (123), the ratios of certain leukocyte populations in fish blood can provide insights into the response to defined treatments or environmental variables. Stress hormones inhibit the proliferation of lymphocytes (58), the apoptosis of granulocytes (124), and the emigration of monocytes and neutrophilic granulocytes from the hematopoietic tissue of the head kidney into the peripheral blood (125). A high number of circulating leukocytes (leukocytosis) can therefore reflect an increased number of monocytes (monocytosis) and neutrophilic/heterophilic granulocytes (neutrophilia) and a concomitant decrease in the number of lymphocytes (lymphopenia) (126). The resulting dysregulation of the immune system can lead to a persistent inflammatory state in fish (127).

Although the neutrophil/heterophil-to-lymphocyte ratio is considered as an important indicator of distress across vertebrate species, it does not necessarily correlate with stress-hormone levels (128). The delayed mobilization of immune cells following the cortisol peak has been regarded as a mechanism that enables the immune system to respond once the primary threat has been overcome (129). The response of the neutrophil/heterophil-to-lymphocyte ratio to long-term environmental stressors is detectable over relatively long periods of time, in contrast to the temporary increase in hormone levels (130). Infections also have a decisive influence on the proliferation and trafficking of leukocytes (131), and the coincidence of stress and immune responses can have an antagonistic effect on the ultimate number of peripheral leukocytes (132).

Differential blood counts have been used to assess the effects of acute and chronic stress events, such as heavy metal exposure in common carp *(Cyprinus carpio)* (133), organochloride herbicide exposure in African catfish (*Clarias gairepinus*) (134), or crowding in Atlantic salmon (*Salmo salar*) (135). Nevertheless, conducting a differential blood count in fish is not very common in clinical laboratory diagnostics, often because reference values are missing.

Flow cytometry is an alternative method for studying the blood cell composition and has the advantage of high sample throughput. In the flow cytometer, individual blood cells successively pass by a laser beam, and the light they scatter is characteristic for a specific blood cell population, allowing their separation. The use of specific antibodies facilitates a more precise determination of the proportions of specific immune cell subpopulations in the blood. Fish cell sorting has depended more on cell characteristics, such as size and granularity, because of a general lack of fish-specific antibodies except for a few model fish species (7, 136).

Similar alterations in blood cell composition have been studied in different fish species exposed to various types of stress (137). For instance, in channel catfish (*Ictalurus punctatus*), the number of circulating neutrophil granulocytes increased after transportation stress (138), while the overall number of leukocytes (including lymphocytes) decreased. Another study on *I. punctatus* confirmed the occurrence of the previously observed neutrophilia simultaneously with decreasing numbers of peripheral B-lymphocytes in response to handling and transportation (Table 1) (65). In Gulf killifish (*Fundulus grandis*) and sea trout (*Cynoscion nebulosus*), the response to crude-oil pollution was characterized by a significantly decreased number of circulating lymphocytes and an increased number of monocytes and eosinophilic granulocytes, respectively (139). Maraena whitefish (*Coregonus maraena*) exposed to high stocking densities (100 kg/m^3^) showed strong increases in the numbers of myeloid cells (granulocytes, monocytes, myeloid dendritic cells, and mast cells), whereas the number of thrombocytes was significantly reduced (Table 1) (32). Overall, different types of stress apparently trigger an increased mobilization of myeloid cells and a reduction in the levels of lymphocytes in the circulation of the affected fish.

## Hematocrit Measurements

As outlined in the previous section, the stress-related secretion of cortisol might provoke contractions of the fish spleen to induce the release of stored erythrocytes into the peripheral blood (114). This would then elevate the volume percentage of erythrocytes, also referred to as the hematocrit value. Along with the hemoglobin (Hb) content and the leukocyte count, the hematocrit is regarded as a key indicator of the secondary stress response. The hematocrit measurement is easy and relatively inexpensive, as the collected whole blood is simply centrifuged in heparinized micro-hematocrit capillaries that are then read off a measuring template (140). A substantially greater effort is required to establish leukocyte profiles; consequently, hematocrit measurements are about 50 times more common in fish studies, as evident from our recent literature search in the Web of Science.

One important aspect for fish physiologists is the association between the hematocrit and the blood viscosity (141, 142). Wells and Weber measured the blood viscosity in *O. mykiss* kept under stressful conditions and found that a 30% hematocrit indicates an optimal relative oxygen transport capacity in the presence of a blood viscosity with a variable hematocrit but a constant Hb concentration (142). The hematocrit in *S. salar* ranges between 44 and 49% (143); however, the levels depend on the temperature (115, 144), fish strain (145), diet (91), and body weight. Large, active fish generally have a high muscular oxygen demand, which can lead to a stimulation of erythropoiesis in the head kidney (146, 147). Accordingly, the physiological hematocrit differs between fast-swimming pelagic fish and fish living sedentarily or in benthic habits (148).

Anesthesia can increase the hematocrit (149), while malnutrition, infection, or environmental toxins can reduce hematocrit and Hb values in fish (150). Non-physiologically low hematocrit values are considered hallmarks of anemia, a specific pathophysiological stress response. Anemia in fish can be easily diagnosed by examining the gills, although more detailed blood analyses help to identify the cause of anemia (53). A significantly reduced hematocrit and an increased erythropoietin production, for instance, can be observed with experimental hemolysis induced by the hemolytic compound phenylhydrazine in *S. salar* (151).

The hematocrit and Hb values associated with erythrocyte count can also depend on the electrolyte–water balance of the fish blood (152). Stressed freshwater fish undergo a drop in their plasma sodium concentrations, and this drop activates counter-transporting ion channels on the erythrocyte membranes (66). The rising ion concentration then induces an inflow of water, causing the erythrocytes to swell and increase their binding capacity for oxygen. These responses are accompanied by the release of additional erythrocytes from splenic stores to compensate for the increased oxygen demands.

## Measurements of Osmolality and Ion Contents

The electrolyte–water balance in the body is termed its osmolality. The plasma osmolality and osmotic regulatory capacity are measured with an osmometer, whereas a spectrophotometer can identify the contributing ions; these are predominantly sodium, chloride, calcium, magnesium, potassium, and phosphate (46).

Freshwater fish species are hyperosmotic in relation to their habitat, whereas marine fishes are hypo-osmotic to the surrounding sea. The resulting differential osmotic pressures force teleost fishes to undergo continuous osmoregulation. Water and ions are exchanged primarily *via* the skin, gills, intestines, and kidneys (52, 153, 154), though freshwater and marine fishes have developed different strategies to maintain their internal blood osmolality within narrow limits. Osmoregulation is a persistently energy-intensive process, even in the absence of additional stress. However, since stress hormones control both the hydromineral balance and energy metabolism in fish, variations in the osmolality of the blood plasma, including changes in the ion composition, are part of the secondary stress response (155, 156). In general, aversive conditions decrease the osmolality in freshwater fish and increase osmolality in marine fish (Table 1) (59, 69, 70, 145, 157, 158). The exposure to high nitrite concentrations, for example, caused a significant reduction in sodium and chloride ion contents in the blood of the freshwater species yellow catfish (*Pelteobagrus fulvidraco*) (94). Catching and a subsequent 3-h-long transportation of freshwater roach (*Rutilus rutilus*) and common bream (*Abramis brama*) reduced the level of plasma sodium by one-third (66). By contrast, the osmolality significantly increased in the plasma of the marine Japanese flounder (*Paralichthys olivaceus*) after the acute exposure to air (159).

Adverse environmental conditions (e.g., hypoxia, which is often associated with high ambient temperature) require an increased branchial activity to enhance the uptake of oxygen. In (hypotonic) freshwater, this hyperventilation accelerates the loss of osmolytes. The conflicting demands of osmoregulation and respiratory gas exchange cause an increased oxygen uptake and loss of ions from the plasma in freshwater fishes. This concept of the “osmorespiratory compromise” has been well-researched in salmonids (160, 161) and hypoxia-tolerant and euryhaline fishes (162). The salinity level (162, 163), the species-specific cellular gill architecture (164), and various extreme environmental variables (165) influence the osmoregulatory ability of fishes under stressful conditions and can maintain physiological osmolality (166, 167) and ion levels (168) in response to diverse challenges in freshwater and saltwater species in their native osmotic environments. For example, ion concentrations were unaffected in Pacific hagfish (*Eptatretus stoutii*) exposed to hypoxia (169) or in Gulf toadfish (*Opsanus beta*) stimulated with spill oil (170).

Some fishes compensate relatively quickly for increased ion flux rates within a certain range; therefore, ion concentrations and osmolality can serve as suitable indicators of acute environmental stressors (140). On the other hand, both parameters change less rapidly compared to the dynamics of stress-hormone levels, making this difference advantageous for recording post-stress responses.

## Assessment of the Humoral Immune Capacity

The response to distinct external signals involves the neuroendocrine system and the immune system (171). For decades, researchers have extracted various immune-relevant parameters from fish blood and evaluated their potential as indicators for compromised homeostasis (172). The central question addressed by these studies is the extent that stress and related adaptive responses influence immunocompetence in fish.

An initial test for assessing immunocompetence is to record the bacterial growth rate in blood plasma from treated vs. control fish (173–176). For example, the growth of the bacterium *Aeromonas salmonicida* after a 24-h incubation was significantly enhanced in plasma from *O. mykiss* with impaired immune capacity due to the exposure to high temperature coupled with crowding (177). By contrast, the non-stressed fish clearly showed potent bactericidal mechanisms that depended mainly on the concentration of a range of immunocompetent macromolecules known as humoral factors. These humoral factors consist of antimicrobial peptides, antibodies, and complement components (178) that circulate in the body fluids. Many fish physiologists have therefore examined the activity of these specific humoral factors rather than the general bactericidal activity of the plasma.

The bactericidal enzyme lysozyme and the microbe-clearing complement components are important humoral molecules of the teleostean innate immune defense (179, 180) and are frequently used as non-specific immune markers (181). In fish, lysozyme has a broader activity than its mammalian counterpart, and several complement components are present as multiple isoforms in bony fishes (182). Lysozyme and complement components are mainly synthesized by leukocytes and the liver, respectively (183–185), but they are secreted into the blood to eliminate invasive pathogens and harmful agents. However, the actual concentrations of the multiple immune factors that are synthesized and secreted into the blood of various fishes at different stages of an infection process or during a challenging situation are not known. Therefore, the measured values for supposedly one factor could actually represent multiple variants and should be interpreted with caution.

The activity of lysozyme and the complement system are measured using turbidimetric assays (186), lysoplate assays (187), lysorocket electrophoresis (188), or microplate assays (189). These assays have demonstrated, for example, that acute handling increased the activity of lysozyme in *O. mykiss* (181) and goldfish (*Carassius auratus*) (190). By contrast, lysozyme activity decreased in Siberian sturgeon (*Acipenser baerii*) after heat stress (189), in sheatfish (*Silurus glanis*) after exposure to intense halogen light (191), in Nile tilapia (*Oreochromis niloticus*) after exposure to the pesticide chlorpyrifos (91), and in *O. mykiss* after transport and exposure to chemicals (192, 193) or low stocking densities (85). Subordinate *O. niloticus* individuals had lower lysozyme activity levels than their dominant conspecifics (194). Overall, stress seems to dampen the lysozyme activity in fish (193).

The influence of stress on the complement system is ambiguous. For example, heat-stressed *A. baerii* (189) and acutely stressed *S. aurata* (195) showed increased complement activity, whereas this activity decreased in European bass (*Dicentrarchus labrax*), red porgy (*Pagrus pagrus*), and *S. aurata* exposed to crowding (84, 196–198). The complement gene families are expanded in several fishes (180, 185), and this has often been associated with newly acquired or partitioned functions of the original complement factors. Consequently, complement-involving events are likely to be more complex than indicated by the snapshot provided by a complement test result.

Other parameters have also proven significant for the assessment of fish welfare. These include the coagulation capacity of the blood (71), the antibody titer (83, 96), the phagocytic activity (32, 91), or the oxidative burst (199, 200), including the activities of the myeloperoxidase (201), superoxide dismutase (75, 202), glutathione peroxidase (93, 94), or glutathione reductase (152). Together with the bactericidal activity, these parameters provide valuable downstream information about the effects of stress hormone release on the immune performance under challenging conditions. Hormones are also likely to induce rather subtle changes in the activity of these immune parameters, either daily or seasonally, and between the sexes (203–205). Most of these analyses are carried out *ex vivo* within a limited time after the previous collection. The oxidative burst assay or phagocytic tests can also be performed *in vitro* on cultured cells (206).

## *In vitro* Tests on Primary Blood Cell Cultures

The development of *in vitro* systems has allowed testing of the effect of certain stressors or challenging conditions delivered in defined doses, intensities, time frames, and/or time points. The dominant model systems for primary fish cell cultures are still derived from the head kidney and spleen (207–209); however, many protocols for blood cell cultures from different fish species have been established (210–213). These models are mainly used to investigate the influence of microbial structures/vaccines and viruses on particular immune cascades (212, 213). In addition, blood cells from fish are useful for investigating the influence of drugs or environmental toxins on the cellular homeostasis. For example, low concentrations of a halogenated hydrocarbon (once used as the insecticide lindane) were shown to increase intracellular calcium levels in peripheral blood leukocytes of *O. mykiss*, whereas high concentrations reduced the synthesis of vital cytokines and induced cell death (214). The polycyclic aromatic hydrocarbon 3-methylcholantrene stimulated the proliferation of blood leukocytes from *C. carpio*, but inhibited the lymphocyte proliferation in response to immunostimulants (215). Similarly, the toxin microcystin-LR (produced by cyanobacteria) and bisphenol A (the monomer component of polycarbonate plastics) modulated the proliferation of lymphocytes isolated from the blood of *O. mykiss* (216) and *C. auratus* (217), respectively. Leukocytes from *C. carpio* subjected to the “alkaline comet assay” have been used to assess the genotoxic potential of organic sediment by determining the DNA damage (218).

Apart from these toxicological studies, blood cell cultures are actually not suitable for modeling aquaculture-relevant problems, such as malnutrition or stocking density stress. For this reason, generalizing the data obtained from *in vitro* systems to an entire organism or a population is controversial, as the response to environmental stress is usually systemic and involves complex cellular networks and tissue systems that communicate with each other *via* stress-inducible mediators, such as steroids and amines. Only a few studies have investigated how stress hormones affect events like the *in vitro* proliferation of blood cells (219). The further development of three-dimensional cultures from fish cells (220) will bring *in vitro* data one step closer to their practical use as fish model systems.

## Expression Profiling of Selected Genes in Blood Cells

Quantitative PCRs (qPCRs) detect the smallest alterations in the expression of genes (221) that are subject to modulation by environmental challenges or stressors. Importantly, gene expression profiles rarely allow absolute statements about the functioning of biological systems, for a number of reasons. One is that many genes are not completely switched on or off in response to a specific treatment or under certain environmental conditions. Instead, most treatments induce a stronger (upregulated) or a reduced (downregulated) expression of a distinct set of genes, and these expression changes only become evident when treated cells are compared with an untreated matching control. One case in point is the expression of potential thermal indicator genes that correlate with the well-studied response of many fish to suboptimal water temperatures (189). Heat stress is well-known to induce the expression of certain heat-shock protein (HSP) genes, such as *HSP70* (*HSP1A1*) and *HSP90* (*HSP90AA1*) (189, 222, 223), whereas hypothermia also induces the copy number of *HSP90* in the blood of *C. carpio* (224). The HSP-encoding transcripts have also been proposed as indicators of the potentially destructive effects of environmental toxins and pollutants. For example, the level of *HSP90* copies dropped almost by half in the blood cells of *C. carpio* exposed to cadmium for 24 h (224). By contrast, the abundance of *HSPA8* copies increased, together with the *HSP70* level, in silver sea bream (*Sparus sarba*) exposed to sublethal concentrations of cadmium for only 2 h (225).

Several investigations have also demonstrated that thermal stress modulates the expression of immune genes in the blood cells of different fish species. In particular, cytokine-encoding transcripts appear to mirror the immune status during stress (226). The classic cytokines, such as interleukins (IL), tumor necrosis factor α (TNF), interferon (IFN), and transforming growth factor (TGF), are relevant in this context (72, 226, 227). The head kidney, spleen, and liver are the usual tissue choices for quantifying immune-relevant transcripts in stimulated or stressed fish, whereas the skin, gills, and blood are used to detect impaired homeostasis. For instance, a temperature study exposed Atlantic cod (*Gadus morhua*) to water temperatures rising from 10°C to 16°C or 19°C and reported slightly increased plasma glucose and cortisol levels (73). In parallel, upregulated expression was observed for the genes encoding interleukin-1β (*IL1B*), β2-microglobulin (*B2M*), major histocompatibility complex class I (*MHC-I*), and immunoglobulin M light chain (*IGL*) in leukocytes of the thermally challenged *G. morhua* (Table 1). These findings were partly confirmed by a report of increased IgM-transcript levels in blood cells of orange-spotted grouper (*Epinephelus coioides*) after heat shock (77). The Indian major carp (*Catla catla*) exposed to temperatures above and below the optimum temperature of 25°C showed a significant increase in blood expression levels of immune genes coding for toll-like receptors (TLR2,−4,−5) and nucleotide binding oligomerization domain containing proteins (NOD1,−2) (Table 1) (74).

An expanded set of immune genes was profiled in *C. catla* exposed to an oxygen saturation below 3 ppm for 1 h (79). The increased expression of the genes coding for the transcriptional regulator high-mobility-group-box-1 protein HMBG1, the receptors TLR4 and NOD1, and their associated adapter proteins myeloid differentiation primary-response protein 88 (MYD88) and receptor interacting serine/threonine kinase 2 (RIPK2), as well as the cytokines IL6, CXCL8, and IL10 suggested an activation of early innate immune mechanisms by hypoxia (Table 1). Other hypoxia studies reported that considerably fewer genes were regulated in fish blood cells, and most were downregulated. For example, a 1-h exposure of *S. aurata* to hypoxic conditions with an oxygen saturation of 1.3 ppm increased hematocrit, Hb content, glucose and lactate levels, but the level of uncoupling protein 2 (*UCP2*) transcripts in the blood cells was strongly decreased (Table 1) (80). Another research group also investigated *S. aurata* under similarly acute hypoxic conditions but with an extended set of qPCR assays (81). They reported a significant downregulation of *UCP2* along with reduced levels of transcripts coding for antioxidant enzymes (*GST3*), outer and inner membrane translocases (*TIM44* and *TIM10*), respiratory enzyme subunits (*SCO1* and *NDUFAF2*), and also markers of mitochondrial dynamics (*MIRO1a*) and fatty acid β-oxidation (*ACAA2*) (Table 1). A subsequent experiment by the same group exposed *S. aurata* to a lowered oxygen saturation of 2.3 ppm combined with crowding (82). The slightly higher oxygen concentration (compared with the previous hypoxia experiment) or an antagonistic effect of hypoxia and density stress was proposed as reasons for the unexpected lack of modulation of either *UCP2* or 42 other profiled genes (Table 1). The exception was *NDUFAF2* (Table 1), which codes for an assembly factor of the Nicotinamide Adenine Dinucleotide - Hydrogen (NADH): ubiquinone oxidoreductase complex and had been included in the list of differentially regulated genes in their previous hypoxia experiment.

Gene profiling can significantly extend the list of stress response parameters beyond hematological, immunological, and metabolic types by identifying negative biomarkers whose values drop below the control levels (39, 80). A key advantage of gene profiling over the detection of stress hormones is that the stressful events that occur immediately prior to sampling (e.g., due to the capture, stunning, and killing of the animal) are usually not reflected in altered transcript levels, while the levels of ACTH or cortisol increase promptly (see section Immunoassays and Clinical Test Kits). Stress hormones are stored as preformed molecules that can be released within seconds, whereas several minutes are required for activation of the appropriate signaling pathways that culminate in the activation and nuclear transfer of the respective transcription factors that initiate the gene transcription process (228). Nonetheless, stress is mechanistically defined by a hormonal response (229), and this response cannot be adequately demonstrated at the transcript level. Without accompanying data on stress hormone levels, alterations in gene expression might only reflect the adaptive changes in the pathways that reestablish homeostasis.

A shortcoming of transcript-specific assays is that the selection of supposedly suitable parameters is left to the skill and knowledge of the experimenters. Studying the complete set of transcripts facilitates the identification of novel indicators that may not previously have been recognized as relevant. However, in truth, exploratory omics approaches are less effective in elucidating mechanistic insights than they are in generating hypotheses on how subsequent experiments can validate a selection of meaningful biomarkers.

## Blood Transcriptomics

High-throughput transcriptomic approaches, such as microarray or RNA-sequencing (RNA-seq) analyses, allow monitoring of the transcriptional changes in a comprehensive panel of potential indicators for a defined research setting (230, 231). In this way, transcriptomic approaches help to arrange traditional and novel parameters into virtual pathways and/or gene networks. RNA-seq is certainly the transcriptomic method of choice over microarrays, which have only been available for a few fish species for decades (230, 232, 233). This shortage is unlikely to be overcome in the future since the availability of reference genomes from a constantly increasing number of fish species and the reduced costs of deep sequencing have now made RNA-seq analyses highly attractive. Another benefit is that RNA-seq analysis distinguishes between individual transcript variants and ohnologous genes, whereas standard PCRs and microarrays typically do not.

The blood cells of most fish species are nucleated (234), whereas the mature erythrocytes and thrombocytes of mammals lack nuclei. This fact alone makes teleostean erythrocytes and thrombocytes highly interesting for (comparative) transcriptomic analyses to record the constitutively expressed transcripts in either cell type (8, 231, 235). Moreover, erythrocytes have been proven to actively supplement allostatic reactions by the induced expression of certain genes. Most investigations have focused so far on the immune responses of erythrocytes after stimulation with pathogen-associated microbial patterns (236), bacteria (237), or viruses (238, 239).

Beyond this, the impact of only a few other stressors has been investigated on the transcriptional response of blood cells. Following an acute exposure of *O. mykiss* to a 25°C water temperature, the erythrocytes showed altered (at least 2-fold) expression of 26 genes at 4 h and 33 genes at 24 h (240). The panel of upregulated genes comprised a cluster of molecular chaperones, including the genes coding for HSP70 (constitutive and inducible forms), HSP90, and the heat-shock factor-binding protein HSBP1. The genes *HSP90, HSP70*, and zinc-finger AN1-type-containing protein 2B (*ZFAND2B*) were later confirmed by literature-mining approaches to represent robust biomarkers for temperature stress in different tissues of salmonid fish (241, 242). Heat stress also increased the transcription of additional stress-related genes, such as stress-induced-phosphoprotein (*STIP1*) and *JUN*, and also immune-relevant features, including NF-κB inhibitor α (*NFKBIA*) and interferon regulatory factor 1 (*IRF1*) in erythrocytes from *O. mykiss*, whereas immunoglobulin-encoding genes were downregulated. This example once again points to the close interdigitation of immune and stress pathways in teleost fishes (171, 243, 244).

High water temperatures are often associated with oxygen depletion as a co-occurring stressor. The schizothoracine fish (*Gymnocypris eckloni*) is an established model for the study of adaptation (245) and hypoxia tolerance (246). A comparison of two *G. eckloni* cohorts exposed to water containing ~8 mg oxygen per liter (normoxia) or ~3 mg oxygen per liter (hypoxia) for 3 days revealed differential expression of about 70 genes in the blood (*q*-value <0.05) of the stressed fish (246). Insulin-like-growth-factor-binding protein 1 (*IGFBP1*) was among the upregulated genes and had previously been identified in the liver of hypoxia-stressed goby fish (*Gillichthys mirabilis*) (247). The differentially expressed genes were assigned to nine Kyoto Encyclopedia of Genes and Genomes (KEGG) pathways, including hypoxia-inducible factor 1-alpha (HIF1α) signaling and fructose/mannose metabolism. Both pathways are expected to be regulated in the context of oxygen depletion in particular and stress in general.

The transcriptome of blood samples from *C. auratus* was analyzed to identify a suitable anesthesia method for routine use in aquaculture (see section Anesthesia and Blood Sampling Procedures) (248). This study revealed that most genes were differentially regulated after percussive stunning (877 at least 2-fold regulated features, *q*-value <0.05), compared with two chemical anesthetics, MS-222 (487 genes) and eugenol (208 genes). Handling of *C. auratus* triggered the upregulation of a large cluster of genes involved in general stress responses (including heat and cold shock, oxidative stress, and endoplasmic reticulum stress), whereas the anesthetized groups showed comparably fewer differentially regulated stress genes. In addition, all three anesthetics effectively maintained the serum cortisol at low levels (<100 ng/ml).

Transcriptomic profiles are critical for understanding relevant functional pathways and networks, but they also have limitations. One serious problem regarding transcriptome analyses of blood samples (and in other samples from whole tissues and organs as well) is that blood is generally a very heterogeneous mixture of cells, so the transcripts represent an average over a broad range of populations. Single-cell RNA sequencing (scRNA-seq) is one step beyond whole-transcriptome analysis, as it identifies the entirety of the transcriptional changes at the level of an individual cell. The use of an scRNA-seq approach in zebrafish provided novel insights into the unique expression patterns of rare immune cell subsets in the teleostean spleen (249) and documented that scRNA-seq created multifold possibilities for recording the tailored response of distinct blood cells to a defined stressor.

## Blood Proteomics

The debate regarding how well-transcript and protein levels correlate (250, 251) is fueled by continuously published confirmatory and contradictory results. Therefore, the safest policy is to consider transcriptome and proteome datasets as complementary. Before qPCR analyses became a standard method in research, antibodies were exploited to record specific biomarkers in blood. The parameters chosen for examination were mostly the already established markers. An additional limitation was that the antibodies, which had generally been produced for mammalian antigens, needed to be cross-reactive (i.e., be able to recognize well-conserved epitope sequences). For instance, the elevated levels of HSP70 in the blood of *O. niloticus* (Table 1) were reported to indicate acute hypoxia (78), whereas the elevated levels of ubiquitin in the erythrocytes of blue maomao (*Scorpis violaceus*) suggested confinement stress (252). The protein analysis conducted by 2D- or differential polyacrylamide-gel electrophoresis or by liquid chromatography coupled to tandem mass spectrometry and using matrix-assisted laser desorption/ionization and time-of-flight-mass analysis, now provides more comprehensive insights into the dynamic allostatic events occurring at the protein level (253).

The effects of handling stress in *S. salar* were examined by Liu et al. (68), who profiled the O-acetylation of sialic acids in the serum. They found that the levels of di-O-acetylated sialic acids increased (Table 1), whereas the levels of mono-O-acetylated sialic acids decreased significantly in stressed fish (68). The exposure of Atlantic halibut (*Hippoglossus hippoglossus*) to an optimal temperature of 12°C and a suboptimal temperature of 18°C in combination with high-CO_2_ water (1,000 matm) for 14 weeks resulted in increased levels of the complement component C3 and fibrinogen γ chain (FGB) in the plasma of both high CO_2_-exposed groups (Table 1) (97). A synthesis of these two factors is triggered very early after injury and pathogen invasion. The plasma of salinity-stressed Mozambique tilapia (*Oreochromis mossambicus*) also showed high concentrations of C3, together with NADH dehydrogenase, Mg^2+^-dependent neutral sphingomyelinase, semaphorin, and caspase-3 (254). The phagocytic activity of leukocytes from the head kidney and spleen also decreased in parallel, suggesting that both aspects might be causally connected.

## Blood Plasma Metabolomics

Metabolome research is increasingly finding its way into aquaculture research, but it still lags behind the metabolomic-based research in mammalian models (46). Most metabolite structures are identical across species, in contrast to gene and protein sequences; therefore, the analytical assays do not need to be customized for a particular investigated species (34). The metabolites found in the blood plasma include various intermediates from a wide range of biochemical pathways. For this reason, metabolomic analyses of blood serum can be used to understand nutritional (89, 90), developmental (255), or pathophysiological (256) aspects of fish physiology and are increasingly being used for disease diagnostics (257). The most commonly used analytical techniques for studying endogenous metabolite profiles are nuclear magnetic resonance spectroscopy in combination with mass spectrometry or vibrational spectroscopy (258). Most metabolomic studies that have been conducted on plasma samples from fish have dealt with toxicological questions (259–262). Application of a five-percent-by-weight concentration of heavy oil has been reported to increase the levels of several plasma metabolites, including amino acids, butyrate derivatives, creatinine, glycerol, and glucose, in *C. carpio* (259). These findings suggested a perturbed tricarboxylic acid cycle of energy metabolism. A similar conclusion was drawn following the analysis of plasma samples from zebrafish exposed to the herbicide acetamiprid (260). The insecticide chlorpyrifos was found to enhance gluconeogenesis (glucose and glycerol), fatty acid metabolism (3-D-hydroxybutyrates and acetoacetate), energy metabolism (creatine), and glutamate generation (glutamine and proline) in *C. carpio* (261). *O. mykiss* exposed to the synthetic contraceptive estrogen ethinylestradiol revealed increased vitellogenin levels, concomitant with significant changes in the plasma lipid profiles that, in turn, were attributed to the high lipid content of vitellogenin (262).

Metabolomic approaches for blood plasma analysis have also been utilized to address aquaculture-related issues. Food deprivation in juvenile *O. mykiss* increased the level of very-low-density lipoproteins while reducing the concentrations of high-density lipoproteins, choline, β-glucose, and lactate, in fasted fish (Table 1) (89). The daily netting of juvenile *S. salar* for 2 weeks disturbed the plasma metabolic balance, as reflected by altered levels of lipoproteins, lipids, lactate, carbohydrates, and specific amino acids (Table 1) (67).

The concentration of a particular enzyme does not necessarily increase or decrease (coupled to an up- or downregulated gene expression) due to varying environmental conditions, though the efficiency in converting certain metabolites may vary. Therefore, metabolic profiling can provide an alternative list of highly sensitive potential biomarkers (34) that can complement the findings of PCR-based techniques and transcriptomics or antibody-based techniques and proteomics. This type of holistic approach can help to coordinate the differentially regulated features in blood and plasma/serum samples in cases where elevated concentrations of a certain metabolite co-occur with increased levels of the associated catalyzing enzyme and with upregulated expression of the enzyme-encoding gene. However, these holistic high-level approaches (cf. Figure 1) remain to be performed in fish.

## Conclusions

Blood contains easily accessible information about the individual physiological state of a fish. Nonetheless, blood is not the appropriate matrix for every research question; for instance, not all aspects of “welfare” can be detected in the blood. Several studies have reported the isolation of steroids from matrices other than blood (i.e., mucus, scales, feces, or water) (263–266); however, the data obtained directly from blood are still far more accurate, as the risk of rapid cortisol degradation and contamination from external cortisol sources are evidently lower (16). Most blood sampling techniques are considered minimally invasive for fish above a given size, though sampling activates primary stress responses within minutes. During the experimental manipulations, the researcher should remain aware that the sampling itself might conceal the hallmarks of a (stress) response to previous treatments, thereby biasing the interpretation of the extracted data. In general, the interpretation of blood-derived parameters requires caution, since particular physiological perturbations do not necessarily depend on a given experimental protocol. The metabolic changes, for instance, might also result from persistent chronic disturbances and/or causally independent events (e.g., circadian rhythms, seasonality, feeding times, conspecific aggressions, water quality, etc.) or substandard sampling and laboratory-specific procedures. The influence of sex and body weight/size of the individual fish should also not be underestimated. Multiple parameters should be recorded simultaneously, preferably from different analysis techniques, to disclose unsuitable husbandry conditions and to identify less obvious or previously unnoticed environmental stressors that exceed the adaptive capacity of fish. This approach supports identification of the comprehensive signature of a distinct stressor, thereby allowing valid conclusions to be drawn regarding fish welfare aspects. Unfortunately, the question of which method(s) should be used to detect the signature of a distinct stressor cannot be answered given the current state of knowledge.

This manuscript reviews different methods for recording welfare-related physiological processes in fish blood. Over the past few decades, a broad repertoire of fish-specific tools and methods has been established that enables the quantification of the concentrations of numerous hormones, metabolites, immune factors, and relevant transcripts that now supplement the panel of traditional biomarkers in blood. In the future, high-throughput -omics technologies (particularly transcriptomics, proteomics, and metabolomics) are expected to provide holistic snapshots of the physiological state of an individual. Assembling the ever-growing number of -omics puzzle pieces will ultimately provide a comprehensive picture of the metabolic, transcriptional, and immunological activities in the blood (and other tissues) of fish. Recent technological innovations, such as scRNA-seq and spheroid cell cultures, will further boost the identification of transcriptional signatures in blood cells of farmed and model fish species.

## Author Contributions

HS wrote sections anesthesia and blood sampling procedures, hematological profiling and blood cell sorting, hematocrit measurements, measurements of osmolality and ion contents, and assessment of the humoral immune capacity. BB wrote sections anesthesia and blood sampling procedures and immunoassays and clinical test kits. AR wrote sections hematological profiling and blood cell sorting, assessment of the humoral immune capacity, *in vitro* tests on primary blood cell cultures, expression profiling of selected genes in blood cells, blood transcriptomics, blood proteomics, and blood plasma metabolomics. All authors wrote the introduction, conclusion, and edited the entire manuscript.

## Conflict of Interest

The authors declare that the research was conducted in the absence of any commercial or financial relationships that could be construed as a potential conflict of interest.

## Abbreviations

ACTH: adrenocorticotropic hormone
CRH: corticotropin-releasing hormone
Hb: hemoglobin
HSP: heat-shock protein
IL: interleukin
KEGG: Kyoto Encyclopedia of Genes and Genomes
MHC: major histocompatibility complex
NOD: nucleotide binding oligomerization domain containing protein
NADH: Nicotinamide Adenine Dinucleotide - Hydrogen
RIA: radioimmunoassay
RNA-seq: RNA sequencing
scRNA-seq: single-cell RNA sequencing
TLR: toll-like receptor.
